# Language Matters: Is There Gender Bias in Internal Medicine Grand Rounds Introductions?

**DOI:** 10.7759/cureus.60573

**Published:** 2024-05-18

**Authors:** Reem M Hanna, Eric Grimm, Angela Keniston, Rafina Khateeb, Areeba Kara, Marisha Burden

**Affiliations:** 1 Department of Medicine, University of Colorado School of Medicine, Aurora, USA; 2 Department of Internal Medicine, University of Michigan Medical School, Ann Arbor, USA; 3 Division of General Internal Medicine and Geriatrics, Indiana University School of Medicine, Indianapolis, USA

**Keywords:** observational study design, leadership, career development, natural language processing, grand rounds, gender bias

## Abstract

Purpose: We performed an exploratory evaluation of gender-specific differences in speakers and their introductions at internal medicine grand rounds.

Method: Internal medicine grand rounds video archives from three sites between December 2013 and September 2020 were manually transcribed and analyzed using natural language processing techniques. Differences in word usage by gender were compared.

Results: Four hundred and sixty-two grand rounds held at three institutions were examined. There were 167 (34.6%) speakers who were women and 316 (65.4%) who were men. The proportion of women speakers was significantly lower than that of women in the internal medicine workforce (34.6% vs. 39.2%, p = 0.04). Among 191 external speakers, only 57 (29.8%) were women. The use of professional titles was equivalent between genders. Despite equal mention of specific achievements in both male and female speaker introductions, there was a trend toward casting female speakers as being less established.

Conclusion: There is a need to adopt processes that will decrease inequities in the representation of women in grand rounds and in their introductions.

## Introduction

Gender disparities within the leadership of academic medicine persist [1]. Equal numbers of women and men now graduate from medical school, yet only 18% of all department chairs are women [2]. Unconscious bias may be one of the drivers of these disparities, creating barriers to the advancement of women [3].

“Grand rounds," or recurring educational events attended by faculty, are an important avenue for increased recognition and for the dissemination of a speaker’s scholarly achievements. Presenting at grand rounds is linked to academic promotion [4]. However, fewer women deliver grand rounds [5], and prior studies have demonstrated that professional titles are less likely to be used for women than men during grand rounds [4,5]. Professional titles convey competence, and failing to acknowledge them can influence the perceived expertise and authority of the speaker [6]. Culturally reinforced stereotypes also contribute to gender disparities in academic medicine. Broad societal stereotypes characterize women as “communal” (e.g., nurturing and kind), while deficient in “agentic” traits (e.g., independent and strong) that are typically associated with men [7]. These stereotypes disadvantage women in historically agentic domains, such as medicine and leadership [8]. The assumption that women with their communal traits will be “less competent and less likely to succeed” than men continues to adversely affect women in academic medicine [8].

While gender differences in the use of professional titles during grand rounds introductions have previously been described, less is known about the use of other phrases or descriptors in speaker introductions that may differ between women and men [4]. We aimed to conduct an exploratory evaluation of gender-specific differences in introductions for speakers across three academic institutions using natural language processing.

## Materials and methods

We conducted a retrospective observational study of internal medicine ground rounds at three US academic sites (University of Colorado, University of Michigan, and Indiana University). Internal medicine ground rounds video archives between December 2013 and September 2020 were reviewed, and introductions were manually transcribed. The gender, using a binary designation of woman or man, for each speaker and introducer (based on gender expression and pronouns used), whether the speaker was internal or external to the site, and the time taken for each introduction was noted, along with professional titles (e.g., "doctor"). If multiple speakers were introduced, information for each speaker was counted as a distinct introduction.

The R package quanteda was used to break up introductions into individual words and two- and three-word phrases [9-11]. Symbols, punctuation, and common words that do not contribute to meaning (e.g., “the”) were removed [9,10]. Words were converted to a shared base word by removing word suffixes (e.g., “awards”/“awarded” to “award”) using the R package lexicon in order to combine conceptually similar words (R package version 1.2.1, 2018). Multi-word phrases were included to help capture additional context that may not be apparent from individual words [9,10]. For instance, the phrase “young career” provides information about the stage of a person’s career that is not available from the word “career” alone. Similarly, precise award types, such as K awards or teaching awards, can be identified using multi-word phrases. Terms with similar meanings were combined based on a review of synonyms queried using the lexical database WordNet via the R package wordnet (WordNet Interface, R package version 0.1-15, 2020). If synonyms had not been combined, each unique word would have been counted separately despite some words having similar meanings. Terms used in only one introduction were removed since they did not provide information about differences in usage. Words and phrases that were hypothesized to differ in usage between introductions of women and men were pre-selected before performing hypothesis tests (Appendix 1). 

Words and phrases were categorized as follows: 1) general descriptors of the speaker, 2) general descriptors of the speaker’s work, and 3) a call-out of specific achievements (e.g., "NIH" and "New England Journal"). Within general speaker descriptors, we further categorized terms that 1) denoted that the speaker was still in early development (e.g., "early career" and "outstanding early"), 2) that the speaker was established (e.g., "international authority" and "national expert"), 3) terms of superlative praise (e.g., "stellar" and "outstanding"), or 4) neutral terms (e.g., "teacher" and "recruit"). Characterizations of the speaker’s work were categorized as being 1) communal (e.g., "contribution") or 2) agentic (e.g., "develop" and "revolutionize") (Appendix 2).

Proportions of women and men were compared to expected proportions using 2021 workforce data available from the Association of American Medical Colleges (AAMC) for internal medicine [12]. We thought that this was an appropriate comparative baseline because the speakers represented many different academic medical centers, in addition to the three institutions being studied. Differences between genders were compared using chi-square or Fisher’s exact tests. Analyses were performed using R version 3.6.3 (R Core Team, Vienna, Austria).

The protocol was reviewed and/or deemed exempt by the institutional review board of each site (Colorado Multiple Institutional Review Board with approval #19-2516, Institutional Review Boards of the University of Michigan with approval #HUM00184424, and Institutional Board of Indiana University with approval #2007579787).

## Results

Over the study period, 462 internal medicine grand rounds were held across the three sites featuring 483 speakers. There were 167 (34.6%) speakers who were women and 316 (65.4%) who were men. The proportion of total women speakers was statistically significantly lower than the proportion of the internal medicine physician workforce who are women (34.6% vs. 39.2%, p = 0.04). There were 110 unique introducers, of whom 24 (21.8%) were women and 86 (78.2%) were men. Of all speakers, 191 (39.5%) were invited speakers external to the site. Among invited speakers, 57 (29.8%) were women, while 134 (70.2%) were men (Table 1).

**Table 1:** Demographics of internal medicine grand rounds introduction Note that there were only 110 unique introducers as some introduced many grand rounds.

	Speaker gender		
	Women (n = 167)	Men (n = 316)	Total (n = 483)
Speaker location, n (%)			
Site 1	25 (15.0%)	53 (16.8%)	78 (16.1%)
Site 2	66 (39.5%)	112 (35.4%)	178 (36.9%)
Site 3	76 (45.5%)	151 (47.8%)	227 (47.0%)
Internal/external speaker, n (%)			
Internal	109 (65.3%)	180 (57.0%)	289 (59.8%)
External	57 (34.1%)	134 (42.4%)	191 (39.5%)
Missing	1 (0.6%)	2 (0.6%)	3 (0.6%)
Gender of the introducer, n (%)			
Woman	21 (12.6%)	32 (10.1%)	53 (11.0%)
Man	146 (87.4%)	284 (89.9%)	430 (89.0%)
Length of introduction in seconds, mean (SD)	112.8 (46.8)	112.9 (48.9)	112.9 (48.1)

Professional titles were equally likely to be used in introducing men (n = 154, 48.7%) and women (n = 80, 47.9%) (p = 0.94). When men introduced women, the speaker’s full name was used more frequently (n = 116, 79.5%) than when men introduced men (n = 193, 68.0%) (p = 0.017).

Our findings suggest that terms indicating early career development were used more often for women (37.1%) than for men (30.7%) while terms introducing the speaker as an established entity were used more often for men (70.9%) than women (61.7%). These differences did not reach statistical significance (p > 0.05 for both comparisons). Terms calling out specific achievements were used in 61.7% of introductions for women and 57.9% of men (p = 0.48). Examples of specific achievements mentioned included references to federal funding and publications in high-tier journals, such as the Journal of the American Medical Association (JAMA), with no statistically significant differences noted by gender. Mentions of K awards and numbers of publications were more frequently noted in introductions of women speakers. Terms of praise, neutral, agentic, and communal phrases were similarly distributed with no statistically significant differences between the two groups (Table 2). P-values from chi-square or Fisher’s exact tests for each term are shown in Appendix 2.

**Table 2:** Comparison of terms used during introductions by gender A p-value of less than 0.05 was considered significant.

Term category	Number (%) of introductions for women with term (n = 167)	Number (%) of introductions for men with term (n = 316)	P-value
General descriptors of the speaker			
In development	62 (37.1%)	97 (30.7%)	0.18
Established	103 (61.7%)	224 (70.9%)	0.050
Superlative general terms	62 (37.1%)	118 (37.3%)	1.00
General descriptors of the speaker’s work			
Neutral terms	66 (39.5%)	126 (39.9%)	1.00
Communal terms	11 (6.6%)	23 (7.3%)	0.92
Agentic terms	102 (61.1%)	174 (55.1%)	0.24
Specific achievements	103 (61.7%)	183 (57.9%)	0.48

## Discussion

Our study found that (1) women continue to be underrepresented as grand rounds speakers and even fewer are invited as external speakers, (2) women are more often introduced with words that indicate that they are less established despite similar descriptors of specific achievements as men, and (3) the use of professional titles, terms of praise, neutral, agentic and communal phrases, and the duration of the introduction are similar for women and men.

Previous studies that highlighted disparities in grand rounds focused on the lower numbers of women speakers and the gender differences in the use of professional titles during introductions [4,5]. This multi-site investigation confirmed the persistence of disparities in the numbers of women versus men speakers but found no differences in the use of professional titles during introductions. While the more consistent introductions between genders are encouraging and may be explained by increased awareness, the disparities in speaker numbers by gender are concerning. One prior investigation found an average of 17% fewer women were presenting at medical grand rounds than men [5]. In our sample, 30% fewer women were presenters, confirming this gap. In addition, the proportion of women presenters was lower than the proportion of women physicians in internal medicine, with even fewer women represented among external speakers or as introducers. Among the invited speakers across the three institutions, less than a third were women.

Previous research has shown that women lose ground at each rung on the promotion ladder [1]; although roughly equal numbers of women and men become physicians, fewer women may be progressing far enough up the academic ladder to be selected for or invited to give grand rounds. This is likely due to multiple factors, including gender bias, fewer women mentors, hierarchy within the academic environment, and challenges with a family-work life balance [13,14]. Academic promotion criteria often require evidence of a regional or national reputation, and one of the most common accomplishments cited as evidence for this is a track record of speaking at grand rounds and being invited to grand rounds at external sites. Thus, ensuring that women have the same opportunities as men to present their research is imperative to ensure women are promoted at the same frequency as their male colleagues. Interventions have been described that may mitigate these inequities including "open call" processes, which allow self-nomination [15], the use of criteria-based checklists, incorporating diverse nomination committees into the selection processes, and creating speakers’ bureaus [16].

While the use of professional titles was similar between women and men with similar descriptors of achievement, we did note interesting trends in the language used where women were more often introduced with words and phrases that might suggest that their career was less established. By undermining the expertise of women during introductions, the introducers risk activating the audiences’ unconscious biases. Stereotypes can be activated by seemingly trivial cues and adversely impact the stereotyped group -- phenomena described as stereotype "priming" and stereotype "threat," respectively. These descriptors might also be viewed as microaggressions that either consciously or unconsciously continue to marginalize women in the field. Interventions that attempt to mitigate these discrepancies, such as incorporating a script into introductions with the aim of achieving consistency in the use of formal titles and descriptors, could address this. In addition, incorporating unconscious bias and anti-sexist training may help to address these potential activators of bias [17].

Addressing gender disparities in academic medicine is instrumental to promoting equity in the workplace. Studies show that women often feel that their work is less valued than their male counterparts and often receive fewer nominations for leadership positions [18]. These biases impede the retention and advancement of women in academic medicine and shape the experience of many women in medicine [3,8]. By inviting women to be grand rounds speakers and by acknowledging their careers accurately with equivalent language and descriptors, we hope to decrease the feeling of marginalization that many women feel in medicine.

Our study provides unique and novel insights into implicit bias in grand rounds introductions using natural language processing. One benefit of natural language processing is that a large amount of data can be processed and analyzed to better understand if biases or discrepancies are present. As evidenced by this study, natural language processing can be used as an efficient and effective tool to detect gender biases in introductions.

Study limitations

Our study only included grand rounds conducted in internal medicine, and our findings may therefore not be applicable to non-internal medicine disciplines. Speaker selection and introduction practices at study sites may not reflect those adopted by others. We used the binary gender model in our data collection rather than directly asking the speakers and introducers. At least 1.2 million people in the US identify as non-binary and 1.6 million as transgender, therefore, we likely misidentified some individuals [19]. Future research needs to be more inclusive. In addition, we did not capture the academic rank of speakers at the time of their presentation which may have impacted perceptions, but rather chose to focus on specific accomplishments to signal experience and expertise. Opportunities for future studies might include looking at academic rank of speakers at the time of their presentations. In addition, given our interest in identifying patterns, we looked at categories rather than individual terminology used by speakers; thus, we chose not to adjust for multiple comparisons. Nevertheless, the goal of this exploratory evaluation was to inform future work and interventions.

## Conclusions

Gender disparities in speakers and their introductions at internal medicine grand rounds persist. Women continue to be underrepresented as speakers, and while professional titles were used equally, we found a trend where women were less often described as being established despite equivalent descriptors of specific achievements as men. This study highlights the conscious and unconscious assumptions about gender roles and the persistence of gender gaps in internal medicine grand rounds. These exploratory findings provide information for future directions and a starting point for interventions to address important factors that are negatively shaping the experience of many women in medicine. By systemically addressing these biases, we hope to build an inclusive and supportive environment for both men and women to succeed.

## Appendices

Appendix 1

Pre-selected words and phrases that were hypothesized to differ in usage between introductions for speakers who are women vs men are shown below.

Words/phrases expected to be more likely used to describe speakers who are women: "children," "grandchildren," "teachers," "teaching," and "young career."

Words/phrases expected to be more likely used to describe speakers who are men: "amazing accomplishments," "very accomplished," "revolutionized," "define," "create," "develop," "incredibly talented," "stellar," "well-published," "successful," "expert," "outstanding," "spectacular career," "recruited," "nationally-recognized," "funded by NIH or supported by NIH," "funded by AHRQ, JAMA, NEJM (New England Journal of Medicine)," "publications," "contribution," "awards," "AOA (alpha omega alpha)," "RO1, K23, R21, and papers."

Appendix 2

**Table 3:** Distribution of specific terms and domains by gender A p-value of less than 0.05 was considered significant.

Term category	Number (%) of introductions for women with term	Number (%) of introductions for men with term	P-value
General descriptions of the speaker			
In development	62/167 (37.1%)	97/316 (30.7%)	0.18
young	57 (34.1%)	91 (28.8%)	0.27
young career	7 (4.2%)	2 (0.6%)	0.0098
young physician	0 (0.0%)	2 (0.6%)	0.55
young faculty	6 (3.6%)	6 (1.9%)	0.36
outstanding young	2 (1.2%)	0 (0.0%)	0.12
outstanding early	2 (1.2%)	1 (0.3%)	0.28
young woman	2 (1.2%)	0 (0.0%)	0.12
outstanding early scholar	2 (1.2%)	1 (0.3%)	0.28
young investigator	3 (1.8%)	2 (0.6%)	0.35
early faculty	1 (0.6%)	1 (0.3%)	1.00
early career	2 (1.2%)	5 (1.6%)	1.00
early stage	2 (1.2%)	2 (0.6%)	0.61
develop young	1 (0.6%)	1 (0.3%)	1.00
Established	103/167 (61.7%)	224/316 (70.9%)	0.050
successful	4 (2.4%)	14 (4.4%)	0.38
accomplishment	5 (3.0%)	18 (5.7%)	0.27
internationally	2 (1.2%)	14 (4.4%)	0.11
national expert	0 (0.0%)	4 (1.3%)	0.30
expert	96 (57.5%)	202 (63.9%)	0.20
international authority	0 (0.0%)	2 (0.6%)	0.55
renowned expert	2 (1.2%)	0 (0.0%)	0.12
international	13 (7.8%)	27 (8.5%)	0.91
international expert	1 (0.6%)	2 (0.6%)	1.00
internationally know	0 (0.0%)	2 (0.6%)	0.55
national recognition	1 (0.6%)	2 (0.6%)	1.00
nationally	9 (5.4%)	15 (4.7%)	0.93
highly successful	1 (0.6%)	1 (0.3%)	1.00
outstanding career	0 (0.0%)	2 (0.6%)	0.55
successful academic career	0 (0.0%)	3 (0.9%)	0.55
successful academic	0 (0.0%)	3 (0.9%)	0.55
Superlative general terms	62/167 (37.1%)	118/316 (37.3%)	1.00
incredibly	2 (1.2%)	19 (6.0%)	0.026
contributor	0 (0.0%)	4 (1.3%)	0.304
child	10 (6.0%)	9 (2.8%)	0.15
stellar	12 (7.2%)	17 (5.4%)	0.55
outstanding	52 (31.1%)	93 (29.4%)	0.78
outstanding physician	1 (0.6%)	2 (0.6%)	1.00
outstanding clinician	1 (0.6%)	2 (0.6%)	1.00
incredibly good	1 (0.6%)	2 (0.6%)	1.00
excellent teacher	0 (0.0%)	3 (0.9%)	0.55
teach	12 (7.2%)	41 (13.0%)	0.075
teacher	6 (3.6%)	13 (4.1%)	0.97
outstanding teacher	0 (0.0%)	2 (0.6%)	0.55
recruit	46 (27.5%)	86 (27.2%)	1.00
outstanding physician scientist	1 (0.6%)	1 (0.3%)	1.00
quite successful	0 (0.0%)	2 (0.6%)	0.55
Neutral terms	66/167 (39.5%)	126/316 (39.9%)	1.00
contributor	0 (0.0%)	4 (1.3%)	0.30
child	10 (6.0%)	9 (2.8%)	0.15
teach	12 (7.2%)	41 (13.0%)	0.075
teacher	6 (3.6%)	13 (4.1%)	0.97
recruit	46 (27.5%)	86 (27.2%)	1.00
General descriptions of the speaker’s work and achievements			
Communal	11/167 (6.6%)	23/316 (7.3%)	0.92
contribute	3 (1.8%)	3 (0.9%)	0.42
contribution	8 (4.8%)	21 (6.6%)	0.54
strong contribution	1 (0.6%)	1 (0.3%)	1.00
contribute enormously	1 (0.6%)	1 (0.3%)	1.00
research contribution	0 (0.0%)	2 (0.6%)	0.55
Agentic	102/167 (61.1%)	174/316 (55.1%)	0.24
develop	33 (19.8%)	50 (15.8%)	0.33
develop novel	0 (0.0%)	3 (0.9%)	0.55
publish	53 (31.7%)	88 (27.8%)	0.43
expertise	15 (9.0%)	21 (6.6%)	0.45
accomplish	26 (15.6%)	41 (13.0%)	0.52
outstanding research	3 (1.8%)	1 (0.3%)	0.12
outstanding researcher	3 (1.8%)	2 (0.6%)	0.35
productive	4 (2.4%)	4 (1.3%)	0.46
incredibly important	0 (0.0%)	4 (1.3%)	0.30
outstanding job	4 (2.4%)	5 (1.6%)	0.50
develop clinical	1 (0.6%)	1 (0.3%)	1.00
develop expertise	2 (1.2%)	4 (1.3%)	1.00
outstanding clinical	1 (0.6%)	3 (0.9%)	1.00
revolutionize	0 (0.0%)	2 (0.6%)	0.55
successful program	0 (0.0%)	2 (0.6%)	0.55
create	3 (1.8%)	12 (3.8%)	0.35
Specific achievements	103/167 (61.7%)	183/316 (57.9%)	0.48
NIH	41 (24.6%)	65 (20.6%)	0.37
paper	43 (25.7%)	70 (22.2%)	0.44
K23	7 (4.2%)	3 (0.9%)	0.037
K award	4 (2.4%)	0 (0.0%)	0.014
publish several	4 (2.4%)	1 (0.3%)	0.051
JAMA	7 (4.2%)	7 (2.2%)	0.26
graduate Alpha Omega Alpha	8 (4.8%)	10 (3.2%)	0.52
scientist award	3 (1.8%)	3 (0.9%)	0.42
England journal	9 (5.4%)	16 (5.1%)	1.00
New England journal	9 (5.4%)	16 (5.1%)	1.00
publish paper	1 (0.6%)	1 (0.3%)	1.00
several teach award	1 (0.6%)	1 (0.3%)	1.00
honor award	1 (0.6%)	1 (0.3%)	1.00
publish widely	1 (0.6%)	1 (0.3%)	1.00
win award	1 (0.6%)	1 (0.3%)	1.00
get teach	1 (0.6%)	1 (0.3%)	1.00
get teach award	1 (0.6%)	1 (0.3%)	1.00
publication	20 (12.0%)	37 (11.7%)	1.00
young investigator award	1 (0.6%)	2 (0.6%)	1.00
win many award	3 (1.8%)	6 (1.9%)	1.00
receive many award	2 (1.2%)	5 (1.6%)	1.00
honor journal	0 (0.0%)	2 (0.6%)	0.55
receive many teach	0 (0.0%)	2 (0.6%)	0.55
impact paper	0 (0.0%)	2 (0.6%)	0.55
important paper	0 (0.0%)	2 (0.6%)	0.55
many publication	1 (0.6%)	4 (1.3%)	0.66
scientific achievement award	1 (0.6%)	4 (1.3%)	0.66
Alpha Omega Alpha	21 (12.6%)	42 (13.3%)	0.94
many award	9 (5.4%)	20 (6.3%)	0.83
win teach award	0 (0.0%)	3 (0.9%)	0.55
national award	0 (0.0%)	3 (0.9%)	0.55
teacher award	0 (0.0%)	3 (0.9%)	0.55
win several award	1 (0.6%)	5 (1.6%)	0.67
achievement award	2 (1.2%)	7 (2.2%)	0.73
research accomplishment	0 (0.0%)	4 (1.3%)	0.30
important publication	0 (0.0%)	4 (1.3%)	0.30
several award	1 (0.6%)	9 (2.8%)	0.18
teach award	4 (2.4%)	23 (7.3%)	0.044
